# Single-cell RNA-seq methods to interrogate virus-host interactions

**DOI:** 10.1007/s00281-022-00972-2

**Published:** 2022-11-21

**Authors:** Kalani Ratnasiri, Aaron J. Wilk, Madeline J. Lee, Purvesh Khatri, Catherine A. Blish

**Affiliations:** 1 Stanford Immunology Program, Stanford University School of Medicine, Stanford, CA 94305 USA; 2 Department of Medicine, Division of Infectious Diseases and Geographic Medicine, Stanford University School of Medicine, Stanford, CA 94305 USA; 3 Medical Scientist Training Program, Stanford University School of Medicine, Stanford, CA 94305 USA; 4 Institute for Immunity, Transplantation and Infection, Stanford University School of Medicine, Stanford, CA 94305 USA; 5 Department of Medicine, Center for Biomedical Informatics Research, Stanford, CA USA; 6 Inflammatix, Inc., Sunnyvale, CA 94085 USA; 7 Chan Zuckerberg Biohub, San Francisco, CA 94158 USA

**Keywords:** Single-cell RNA sequencing, Antiviral immunity, Virus, Transcriptomics

## Abstract

The twenty-first century has seen the emergence of many epidemic and pandemic viruses, with the most recent being the SARS-CoV-2-driven COVID-19 pandemic. As obligate intracellular parasites, viruses rely on host cells to replicate and produce progeny, resulting in complex virus and host dynamics during an infection. Single-cell RNA sequencing (scRNA-seq), by enabling broad and simultaneous profiling of both host and virus transcripts, represents a powerful technology to unravel the delicate balance between host and virus. In this review, we summarize technological and methodological advances in scRNA-seq and their applications to antiviral immunity. We highlight key scRNA-seq applications that have enabled the understanding of viral genomic and host response heterogeneity, differential responses of infected versus bystander cells, and intercellular communication networks. We expect further development of scRNA-seq technologies and analytical methods, combined with measurements of additional multi-omic modalities and increased availability of publicly accessible scRNA-seq datasets, to enable a better understanding of viral pathogenesis and enhance the development of antiviral therapeutics strategies.

## Introduction

Viral infectious diseases perennially threaten global health. The most recent pandemic, COVID-19, has globally accounted for more than 584 million SARS-CoV-2 infections and 6.4 million deaths as of August 2022 [1]. Each new viral species, strain, and mutation can influence disease severity through complex host-virus interactions [2–4]. As obligate intracellular parasites, viruses require host cellular machinery for replication. In response, host cells employ antiviral mechanisms to recognize and restrict viral replication [5]. The arms race between virus and host at the single-cell level collectively drives variability within virus and host cell populations, impacting disease pathogenesis and epidemiological dynamics. Untangling intra- and inter-individual heterogeneity in responses to viral infection requires a high-resolution analysis of viral dynamics and the ensuing host response.

**Fig. 1:**
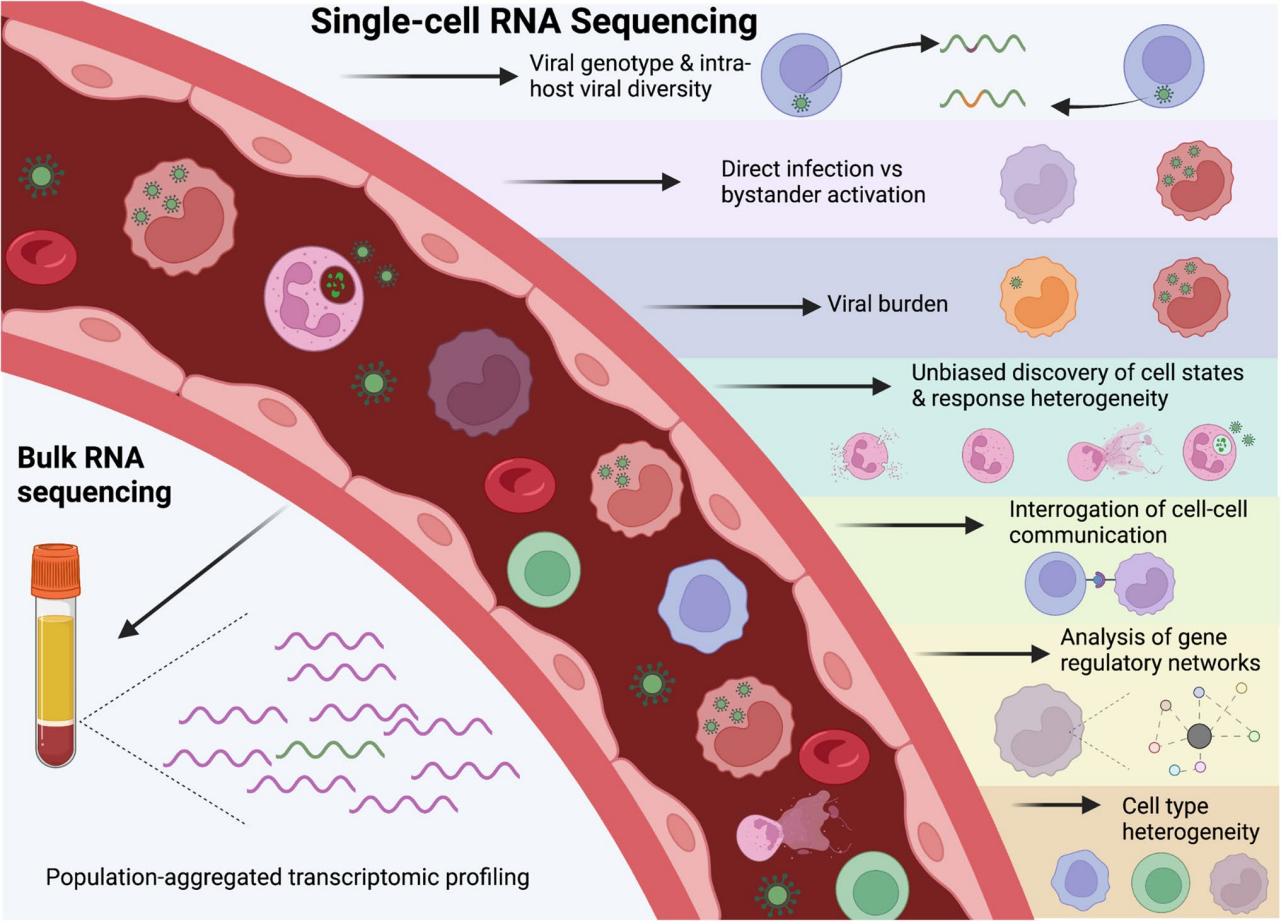
Transcriptomic approaches to profile antiviral immunity

While bulk RNA-seq studies have been instrumental to our understanding of cellular antiviral responses [6–9], these methods average over a population of cells, thereby obscuring underlying heterogeneity. In contrast, single-cell RNA sequencing (scRNA-seq) allows for transcriptome-wide profiling at the resolution of the individual cell, providing a powerful method to interrogate the transcriptomic heterogeneity of cellular responses. This single-cell resolution enables understanding of variations in host responses that can be driven by factors that include direct viral infection versus activation of bystander cells [10–13], viral genotype and intra-host viral diversity [14–17], cell type heterogeneity in disease severity and responses [11, 18–21], and impacts of viral burden on host responses [11, 22, 23] (Fig. 1). scRNA-seq has previously been used to investigate infection by diverse viruses including herpes simplex virus [24, 25], reovirus [26], dengue virus [11, 19, 27], influenza virus [28, 29], HIV [20, 30], hepatitis B virus [10], and SARS-CoV-2 [18, 31–33].

In this review, we aim to provide researchers with tools to inform scRNA-seq study design for understanding virus-host interactions, as well as encourage increased studies across diverse viral species. We highlight various scRNA-seq technologies and methods, from sample processing to computational analyses, and the power and nuances of each in examining antiviral immunology. Throughout, we will present important contributions of previous scRNA-seq studies to our understanding of viral immunology while highlighting areas of scRNA-seq development that show promise. With continued scRNA-seq studies and data sharing, together, this work will expand our understanding of immunological responses across all viruses and drive innovation towards antiviral interventions to combat current and emerging viruses to enhance pandemic preparedness [34].

## Technologies for deep transcriptional profiling of antiviral immunity

### Overview of scRNA-seq workflow

**Fig. 2:**
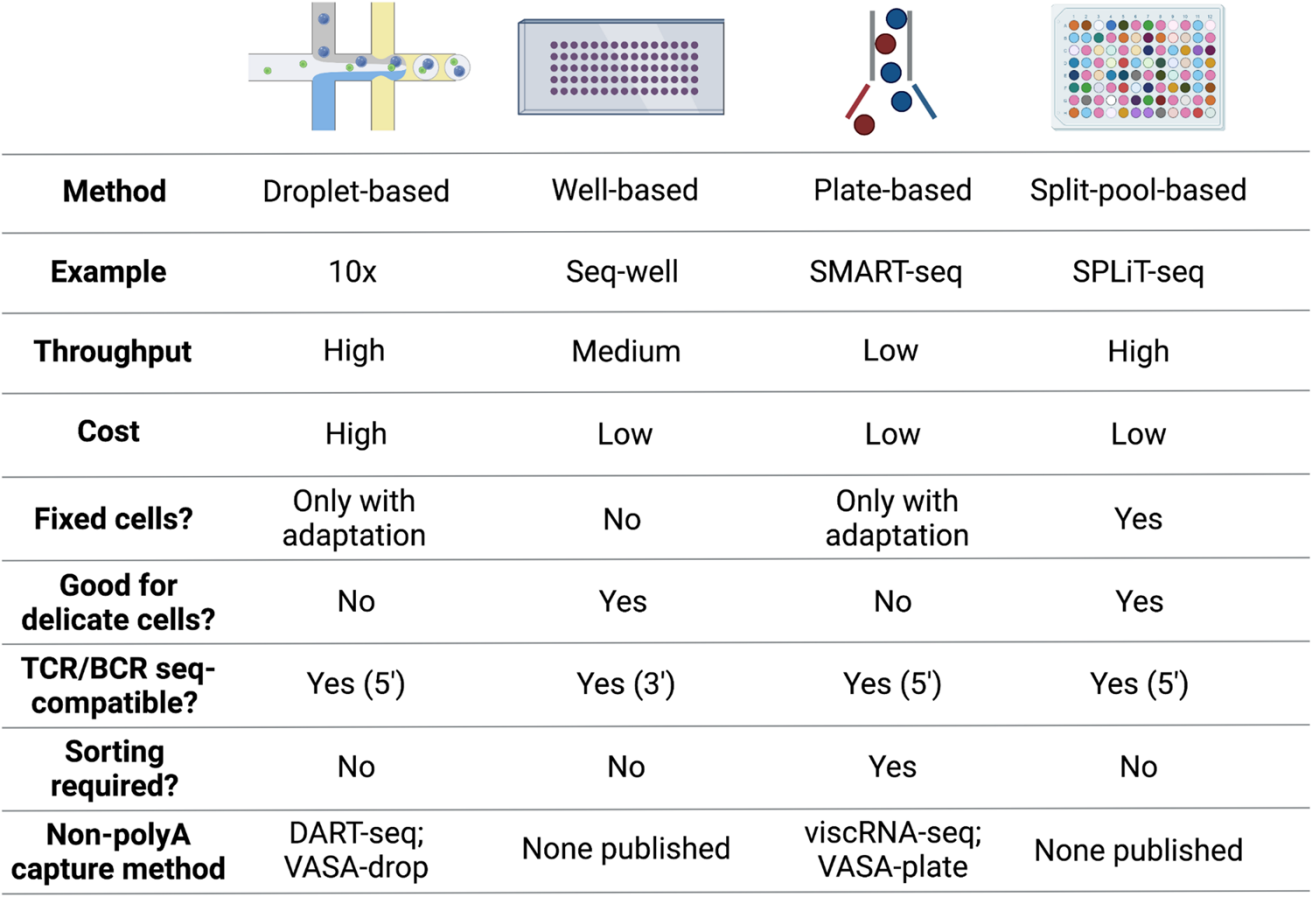
Table summarizing the advantages and disadvantages of different scRNA-seq methods in viral immunology research

The last several years have seen a proliferation of scRNA-seq platforms, each with distinct advantages in scalability, flexibility, applications, and cost. Fundamentally, all scRNA-seq platforms involve the same basic steps: (1) nucleotide barcoding of single cells; (2) cell lysis; (3) capture of mRNA; (4) generation of cDNA through reverse transcription; (5) cDNA amplification by PCR; (6) cDNA library preparation; and (7) sequencing. These methods utilize unique barcoding of each cell to identify each transcript’s cell of origin, with some methods also including unique molecular identifiers (UMIs) which can be added to each transcript prior to library amplification to reduce amplification bias. However, there are important distinctions between the different strategies of performing scRNA-seq which impact each method’s ability to capture transcripts from particular cell types and viruses. Here, we discuss four classes of scRNA-seq technologies that are distinguished by their strategies for single-cell nucleotide barcoding: droplet-based (e.g., 10X), well-based (e.g., Seq-Well), plate-based (e.g., Smart-Seq), and split-pool-based (e.g., SPLiT-seq). In the following sections, we will describe the advantages and limitations of each technology, with a focus on how they can be applied to the study of viral infectious diseases (Fig. 2).

### *Droplet-based methods*

Droplet-based sequencing methods rely on microfluidic mechanisms to encapsulate individual cells with beads bearing cell barcodes in oil droplet emulsions [35]. The use of barcoded beads allows for the identification of transcripts from the same cell during downstream analysis, as each cell co-localizes with one bead bearing a unique oligonucleotide barcode that labels all transcripts in a given droplet. These cells are lysed within these droplets, allowing transcripts to hybridize into the mRNA capture beads. Next, depending upon the platform, reverse transcription can happen either within the droplet or after the demulsification of the droplet. After transcript capture and reverse transcription, standard library preparation procedures occur during which UMI barcodes are added to each transcript prior to PCR amplification. The standard droplet-based platforms include inDrop and Drop-Seq [36, 37], both open-source methods, and the commercially available methods from 10X Genomics Chromium. Differences between these platforms include bead composition, timing of cDNA synthesis, barcode design, and sequence processing. These methods’ power lies in their ease of use and flexibility, with 10X methods particularly increasing the capacity to profile thousands of cells. Recently, 10X Genomics developed the Chromium X controller that pushes high-throughput scRNA-seq sequencing from 10 to 60 K cells per sample by adding additional sensors and more accurate pressure and temperature control during droplet formation. This depth in cellular sequencing enables the profiling of rarer cell states and cell types that may be missed otherwise.

The flexibility and modularity of bead construction have enabled the development and commercialization of multiple different kits on the same platform. For example, beads can be designed to hybridize either the 5’ or the 3’ ends of mRNAs and can include additional primers to enrich for particular transcripts, like B cell receptor (BCR) and T cell receptor (TCR) sequences. Beads can also include oligonucleotides to capture transposed accessible chromatin fragments or antibody-conjugated oligonucleotides, thereby performing multimodal epigenetic or proteomic profiling, respectively. However, this system has difficulty capturing usable transcripts from neutrophils and other granulocytes, even when these cells are freshly isolated, in part due to their high RNAse levels and sensitivity to degranulation during microfluidics processing [38, 39]. While 10X recommends the removal of granulocytes from samples, for those that want to profile these cells, 10X provides recommendations [40, 41] for capturing granulocyte sequences and these have successfully been shown to work on neutrophils [42]. A direct comparison of 10X, inDrop, and Drop-seq found that 10X has the highest molecular sensitivity, highest precision, and least technical noise, though 10X is more expensive and dependent on proprietary reagents [43]. Many studies have leveraged droplet-based methods to analyze thousands of cells across various virus infections [44–48]. Without the constraints of array size, droplet-based methods are highly scalable.

### Well-based methods

Well-based scRNA-seq platforms, such as Seq-Well [49, 50] or the BD Rhapsody platform [51, 52], achieve single-cell barcoding by loading cell suspensions onto microarrays pre-loaded with oligonucleotide-barcoded mRNA capture beads. In Seq-Well, cells are lysed within microarrays sealed with a semipermeable membrane, allowing the RNA from each cell to be captured on an individual barcoded bead and preventing mRNA contamination from neighboring wells. Following capture, the bead-bound RNAs can be released from the arrays and processed for reverse transcription, library preparation, and sequencing.

In addition to being much cheaper than droplet-based scRNA-seq methods, well-based methods have the advantage of being gentler on the cells they process. Importantly, this allows well-based methods to capture sensitive cell types, such as neutrophils, that may be lost during a microfluidic-based workflow. The ability of well-based methods to successfully preserve the transcriptomes of neutrophils has proven invaluable in the study of viral infections, particularly COVID-19 [18, 31, 45]. Seq-Well-based studies have identified key features of neutrophil activation and dysfunction in severe COVID-19 that appear to contribute to disease pathology [18]. Moreover, these studies discovered a population of immature neutrophils that emerges in the peripheral blood of COVID-19 patients that predicts 28-day mortality [18, 31, 45].

The portability and economy of well-based methods have also made it possible to perform scRNA-seq in extraordinary conditions. In a 2020 study, researchers performed Seq-Well on peripheral blood mononuclear cells (PBMC) from non-human primates infected with the Ebola virus under BSL4 conditions [23]. However, the scalability of well-based methods is relatively limited. Additionally, well-based methods are currently limited to use on unfixed cells.

### Plate-based methods

Plate-based methods, such as Smart-seq [53] and MARS-seq [54], rely on cell sorters to place individual cells into individual wells across 96- or 396-well plates. These wells contain lysis buffer and cell barcodes, and can include additional UMIs and plate barcodes. These protocols are amenable to automation with liquid-handling robots and do not require specialized equipment besides a cell sorter and a PCR machine. The Smart-seq protocols are able to capture full-length transcriptome coverage, with methodology improvements made in Smart-seq2 [55] and Smart-seq3 [56] protocols allowing for higher coverage and sensitivity of detected transcripts. The per-plate nature of processing tends to lower throughput (96 or 364 per plate) and technical variability can be introduced in processing steps (e.g., thermocycler used, pipetting steps) that may contribute to batch effects between plate and experiment.

The cell sorting step can be made specific to the inclusion/exclusion of particular cell types (e.g., removal of erythrocytes and doublets, inclusion of specific populations) to thereby be well suited for profiling rarer cell populations. Additionally, using fluorescence-activated cell sorting (FACS) of cells prior to plating allows for cell types to be identified prior to their sequencing, which can help distinguish cell types that are difficult to identify using transcriptomics alone (e.g., intermediate monocytes, NK cells, T cells) and/or gain better sequencing depth into populations that might not be evenly sampled in a pooled scRNA-seq method. A key advantage of these methods is their usefulness in investigating the mechanism and/or transcriptomics of specific cell subsets (e.g., rare cell type, functionally responding cell type, viral-protein expressing cells).

An example of the usefulness of pre-sorting populations comes from Steuerman et al. [29] who FACS-sorted CD45 + (immune) and CD45- (non-immune) populations from the lungs of influenza-infected mice and performed MARS-seq to identify virus and host cell transcripts [29]. This approach enabled sufficient depth of sequencing into both populations to identify multiple immune and non-immune cell types associating with varying proportions of infected cells. While different cell types carried varying levels of influenza transcript load, all infected cells—independent of cell type—demonstrated a conserved transcriptional response marked by repression of mitochondrial-related transcripts. While its strengths lie in pre-sorting to better understand the input cell population prior to sequencing, the sorting associated with plate-based methods can add time and manipulation into sample processing and, paired with the use of fixed plates, can make this method lower-throughput in comparison to droplet-based methods.

### Split-pool methods

Split-pool sequencing is a relatively new technique that leverages combinatorial barcoding to identify individual cells rather than using a physical partition such as a droplet or a microwell [57]. Split-pool scRNA-seq begins by performing reverse transcription on fixed and permeabilized cells to add a sample-specific oligonucleotide barcode to all mRNAs in each sample. The cells are then pooled, redistributed, and barcoded multiple times such that each cell receives a unique combination of barcodes. When performed on a 96-well plate, four rounds of barcoding are sufficient to create over 20 million unique barcode combinations, which can label up to an estimated 1 million cells without creating a significant number of multiplets. Once the four barcoding steps are completed, the cells are lysed and libraries are prepared for sequencing [57].

Split-pool sequencing has the major advantage of needing no specialized equipment. Moreover, split-pool-based platforms are designed to be compatible with paraformaldehyde-fixed samples, making it particularly attractive for researchers working with virus-infected samples that require a high level of biosafety or have cells that can’t be processed immediately (e.g., when thawed for use across multiple experiments). Like well-based platforms, split-pool sequencing technologies are gentle enough for use on delicate cells such as neutrophils that are sensitive to microfluidics. Finally, these methods allow for easy multiplexing of a large number of samples in one experiment.

### Single-cell TCR/BCR sequencing

Technologies relying solely on short-read sequencing of mRNA, while generally appropriate for measuring the abundance levels of most genes, are insufficient for the capture and reassembly of the more complex transcripts encoding the exquisitely specific receptors expressed on T cells (TCRs) and B cells (BCRs). In order to create the extraordinary diversity of TCR and BCR sequences found in humans, DNA segments of the genes encoding these receptors are rearranged in a process called V(D)J recombination. Additional diversity is introduced post-transcriptionally, when the transcripts undergo alternative splicing to produce unique genes that give rise to uniquely specific receptors. Therefore, in order to successfully capture the full diversity of a given V(D)J region, long-read sequencing techniques are necessary. Moreover, while traditional single-cell RNA sequencing approaches capture the 3’ ends of transcripts, the rearranged V(D)J region of a BCR or TCR is located at the 5’ end of the transcript, making it difficult to capture with short-read approaches [58].

In 2019, Repertoire And Gene Expression by Sequencing (RAGE-seq) was developed to pair full-transcript sequencing with 5’ capture in order to robustly resolve TCR and BCR sequences in a high-throughput manner [58]. This method is most commonly used in conjunction with microfluidics-based platforms such as those offered by 10X Genomics, though similar methods have now been developed for compatibility with well-based platforms, including BD Rhapsody and Seq-Well, which use a unique 3’ approach to capture TCR sequences [59]. The advent of techniques that allow for simultaneous study of the whole transcriptome and TCR/BCR sequences provides the opportunity to analyze the transcriptomes of cells with specific TCR or BCRs, for example, clonally expanded populations [60–64].

A limitation of studying antigen-specific T cells is that these populations can be too small to serve as inputs into many scRNA-seq methods. SELECT-seq was developed as a method to address this limitation and gather both TCR sequencing and cellular transcriptomic information for rare/specific cell populations of interest. SELECT-seq does this by using a modified Smart-Seq2 protocol to generate cDNA libraries from single T cells, then taking an aliquot of each library for nested PCR to amplify CDR3 regions of both TCRɑ and TCRβ chains [65]. T cell libraries were selected for further high-coverage in-depth whole transcriptome sequencing based upon whether there were duplicated CDR3 regions present (assumed to be clonally expanded). The authors utilize this method to identify transcriptional differences between CD8 + T cell populations with high versus low clonal expansions. They show that they are able to select for CMV-reactive CD8 + T cell populations (by activating T cells with CMV peptide) and identified that clones with low expansion had increased *IL2RA* (T cell activation signaling receptor) and *CD27* and *CD28* (costimulatory markers) while highly expanded clones had increased expression of *TIGIT* (coinhibitory receptor) and *KLRG1* (senescent marker). The advantage of this method is that it reduces costs associated with whole transcriptome sequencing by allowing users to select for cell populations of interest as well as for specific TCR clones for limited scRNA-seq.

LInking B-cell Receptor to Antigen specificity through Sequencing (LIBRA-seq) can be used to interrogate the antigen specificity of BCRs while simultaneously collecting BCR sequences and whole transcriptome data at the single-cell level. Briefly, LIBRA-seq involves exposing B-cells to a pool of oligonucleotide-barcoded antigens. Antigens bound by a B-cell are then captured within a droplet alongside the cell so that the antigen barcode can be sequenced in conjunction with the B-cell’s mRNA, thereby revealing the specificity of that cell’s BCR [66]. LIBRA-seq has also been leveraged for the discovery of novel broadly neutralizing antibody lineages in HIV-infected patients [66] and the identification of novel neutralizing antibodies against SARS-CoV-2 [67]. It can also be used to interrogate the transcriptomes of viral antigen-specific B-cells [66].

## Adaptations to established scRNA-seq methods for measuring viral transcripts

### Capture of non-polyadenylated viral transcripts

Most scRNA-sequencing methods that perform 3’ mRNA capture (e.g., Seq-Well, BD Rhapsody, Drop-Seq, inDrop, and several 10X Genomics kits) utilize poly-T oligonucleotide (oligo(dT)) primers to capture polyadenylated transcripts associated with human mRNA, while limiting the measurement of highly abundant ribosomal RNAs [68]. Because many viruses are polyadenylated, they can be detected through standard methods: for example, SARS-CoV-2 transcripts can be measured using standard 10X 3’ methods [69, 70] and Ebola virus can be measured using the standard Seq-Well protocol [23].

**Fig. 3:**
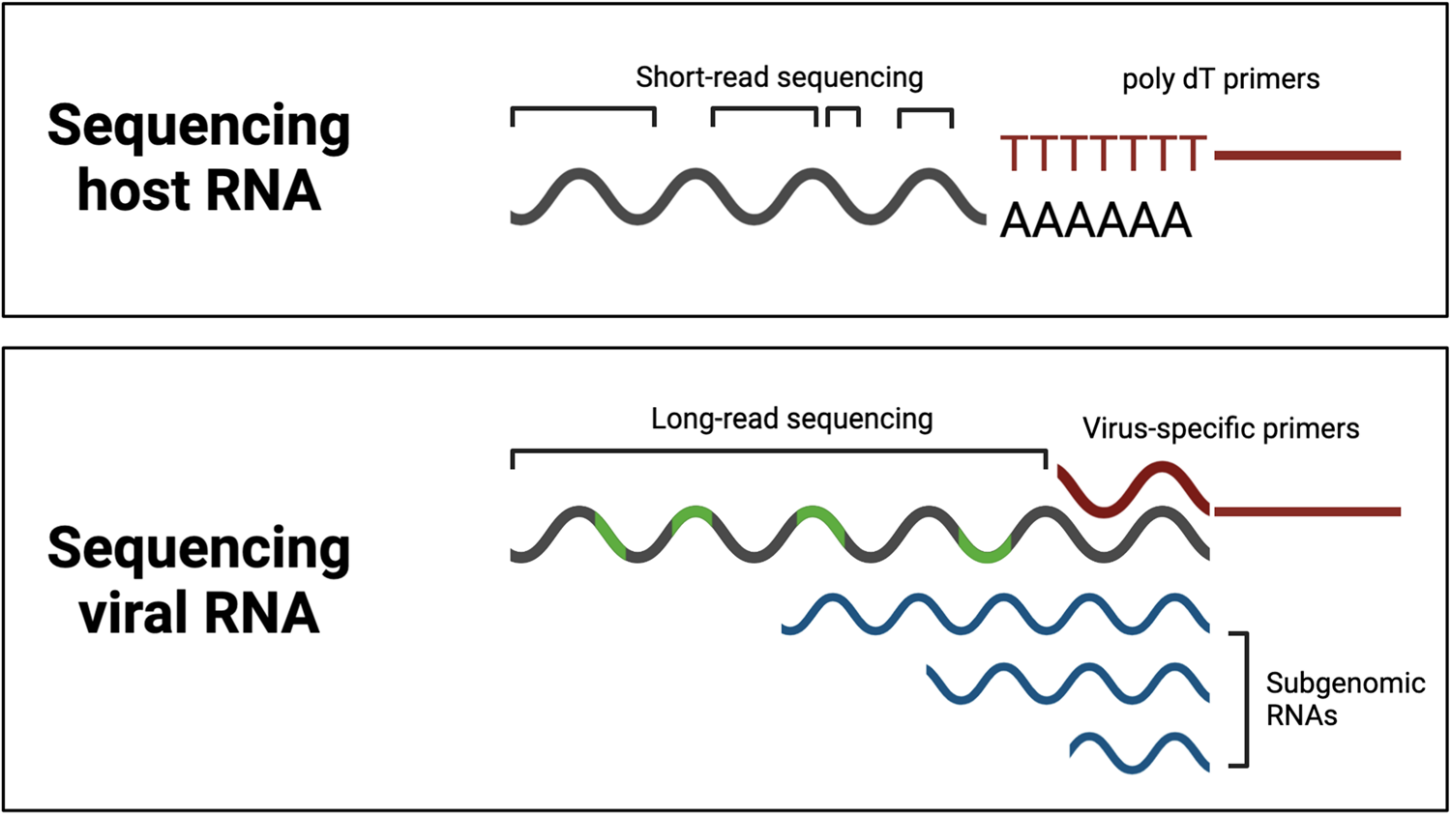
Schematic demonstrating key differences between host and viral RNA that can require alternative approaches for sequencing virus transcripts

However, a limitation of many scRNA-seq methods is that they cannot capture non-polyadenylated transcripts. Some viruses, such as those in the *Flaviviridae* family (e.g., dengue virus, Zika virus, yellow fever virus, and hepatitis C virus), generate non-polyadenylated transcripts. Hence, if the study goal is to understand host responses in relation to viral infection dynamics, it can be important to choose a sequencing method that will capture non-polyadenylated reads. There are numerous methods that have been developed to measure non-polyadenylated transcripts, some of which have been utilized in the context of viruses. For example, virus-inclusive single-cell RNA-Seq (viscRNA-Seq) adapts the Smart-seq2 plate-based scRNA-seq protocol to pair standard oligo(dT) primers with virus-specific primers prior to cDNA generation (Fig. 3). As part of viscRNA-seq, amplified cDNA is split into aliquots for two purposes: (1) qPCR for viral RNA that can be associated with (2) sequencing for host transcripts [11]. This method measured viral RNA transcripts from the dengue virus and Zika virus from in vitro infections and recovered dengue viral transcripts from the PBMCs of dengue patients to identify viral tropism [11].

Droplet-assisted RNA targeting by single-cell sequencing (DART-seq), which is an adaption of Drop-seq to include multiplexed RNA amplicon sequencing, captured the non-polyadenylated viral transcripts in an in vitro reovirus infection [26]. Other methods that utilize intentional primer design to generally capture both polyadenylated and non-polyadenylated transcripts include vast transcriptome analysis of single cells by dA-tailing (VASA-seq) which can be adapted in droplet workflows (VASA-drop) and plate-based workflows (VASA-plate) [71]; single-cell universal poly(A)-independent RNA sequencing (SUPeR-seq) [72]; and multiple annealing and dC-tailing-based quantitative single-cell RNA-seq (MATQ-seq) [73].

### Long-read sequencing for viral genomes

Many RNA viruses have low-fidelity, error-prone polymerases that introduce mutations into the viral genome upon replication. For example, estimates for HIV-1’s RNA-dependent DNA polymerase predict around ~ 5–10 errors per HIV-1 genome per replication round [74]. Thus, during an in vitro or in vivo viral infection, viruses can exist as a viral quasispecies—a population of viruses differing in genetic variation leading to competent and/or defective viral particles that can differentially infect and drive host pathogenesis [73]. scRNA-seq studies that examine host antiviral responses without measuring viral genetic diversity are studying the combined effect of various viral mutations and defects. Most scRNA-seq methods incorporate library fragmentation for downstream short-read sequencing, but viral genomes span from 1–2 kb (Circoviruses) [75] to ~ 30 kb (SARS-CoV-2) and past 1000 kb (Mimiviruses) [76], allowing these methods to only capture a fraction of most viral genomes. Thus, methods that incorporate long-read sequencing empower an understanding of viral genomic diversity in relation to cellular responses in addition to viral transcript abundance (Fig. 3). One such method was developed by Russell et al. which utilizes 10X technology to generate cell-barcoded cDNA from influenza virus (IAV)–infected cells and split the cDNA for two measurements: (1) standard downstream fragmentation and short-read sequencing and (2) enrichment of IAV-specific transcripts through PCR amplification and full-length sequencing using PacBio methods [14]. The group demonstrated that two-thirds of IAV-infected cells had mutations or defects in one or more of IAV’s 7 genomic segments and were able to use this integrated data to identify four IAV defects that correlated with increased cellular IFN induction and further validate these in vitro. An alternative approach, Single-cell Nanopore sequencing with UMIs (ScNaUmi-seq), leverages the 10X Genomics Chromium system to generate cell-barcoded cDNA products that are prepared downstream for Oxford Nanopore long-read sequencing [77].

## Insights from single-cell transcriptional profiling of antiviral immunity

### Unbiased discovery of cell type

**Fig. 4:**
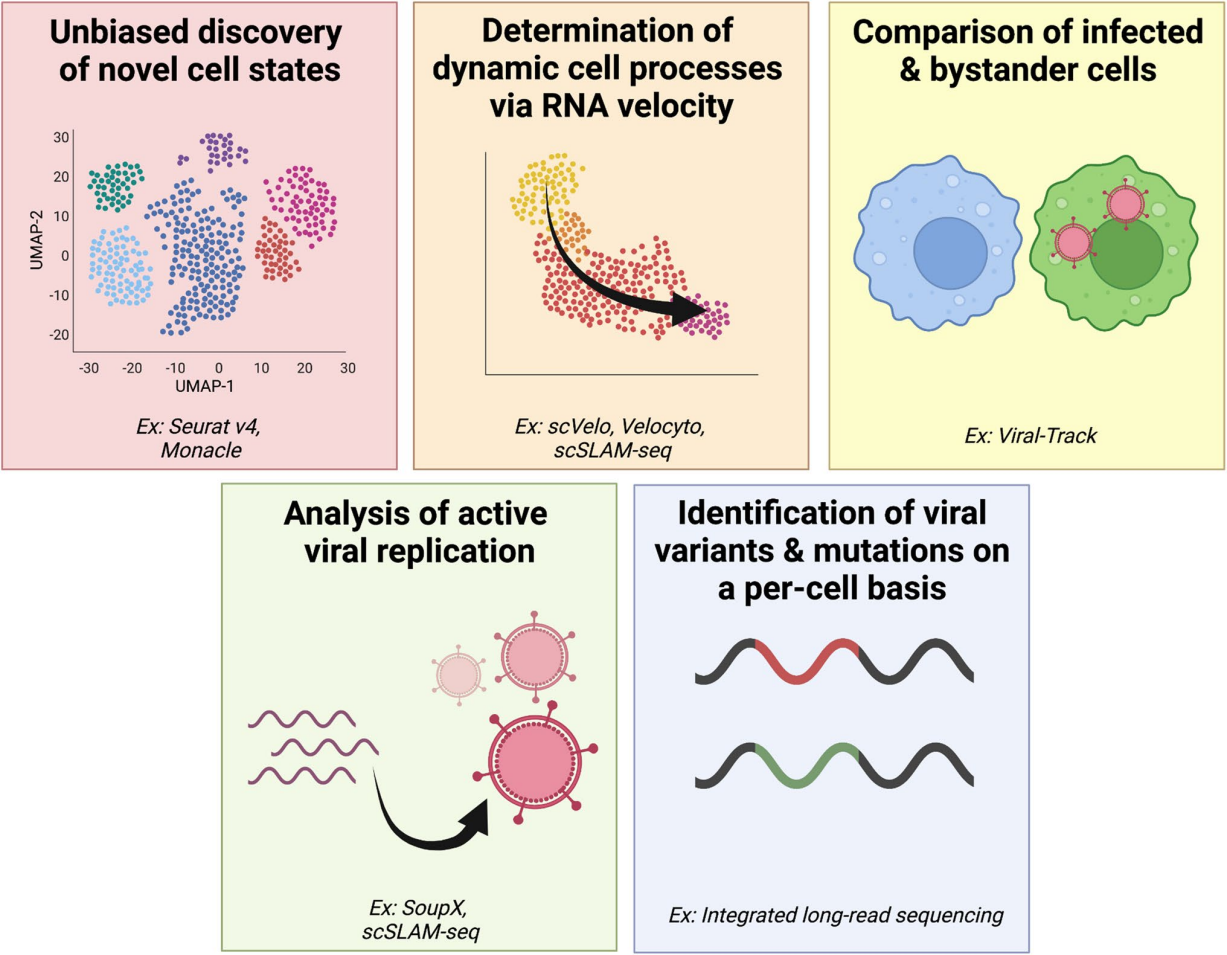
Overview of ways in which scRNA-seq can enable a deeper understanding of cellular antiviral responses and viral dynamics

By profiling cellular phenotype at the transcriptome-wide scale, scRNA-seq enables the discovery of cell type and state that is not biased by the selection of marker panels, as in flow or mass cytometry (Fig. 4). Unbiased discovery of cell type generally relies on unsupervised clustering analysis, which groups transcriptionally similar cells into “clusters” based on gene expression. Most toolkits for scRNA-seq computational analysis, including Seurat, scanpy, and Monocle, include implementations of several graph-based unsupervised clustering algorithms; the strengths and weaknesses of these various algorithms have been extensively assessed and reviewed elsewhere [78–81]. The use of unsupervised clustering to assess cell type composition in scRNA-seq data is advantageous because it does not require a priori knowledge, allowing for easy discovery of unexpected populations.

Analyses of COVID-19 patient samples exemplified the utility of leveraging unsupervised clustering approaches for cell type discovery. Early evidence of emergency myelopoiesis in severe COVID-19 came from scRNA-seq datasets that unexpectedly found immature neutrophils in the peripheral blood of patients with severe COVID-19 [31], which was directly enabled by performing transcriptome-wide measurements. Broadly, cell type annotation in scRNA-seq can be accomplished by manually assigning identities to cell clusters, or by using automated tools to annotate individual cells. In the absence of a suitable transcriptomic reference dataset, differentially expressed genes (DEGs) for each cluster can be compared to known or expected transcriptomic profiles from the literature to call cell types for each cluster [82].

Reference datasets can greatly simplify this task, and a variety of automated tools have been developed to leverage transcriptomic references to annotate query datasets (reviewed by [83]). Generally, these tools operate either by correlating the transcriptomic profile of each single query cell to a reference bulk or single-cell profile (scmap [84], SingleR [85], scMatch [86]), by identifying mutual nearest neighbors between reference and query cells and transferring cell type labels (Seurat v4 [87], scArches [88]), or by performing supervised classification (SingleCellNet [89], scClassify [90], LAmbDA [91], scPred [92]).

Choosing a suitable reference is a critical and challenging step in any automated cell annotation process. The most robust reference-based automated cell type annotations would ideally use references that contain the same cell types as the query and from the same tissue niche. Most reference datasets are derived from homeostatic tissue niches, and care must be taken when interpreting reference-based cell type annotations in a highly perturbed query dataset. For example, there are frequent situations where unexpected cell types are present outside of their typical tissue niche. For example, when emergency myelopoiesis results in immature myeloid cells being present in the peripheral blood [31, 45], applying peripheral blood references to peripheral blood datasets with emergency hematopoiesis [93] will lead to incorrect and misleading cell type annotations [93]. Thus, reference-based cell type annotation should always be paired with manual verification based on biological knowledge and expectations.

In addition to generating and annotating new references, there is an ongoing effort in cell type annotation to incorporate additional data modalities into assigning cell type identity. For instance, T cells contain low RNA content that makes biologically distinct T cell subtypes difficult to distinguish based on transcriptomic data alone [87]. However, these subtypes can be readily dissected by their cell surface proteome. Data from single-cell multimodal methods that capture both transcriptome and cell surface protein data [94] thus have the potential to solve this problem. Recently, Hao et al. [87] introduced weighted nearest neighbor (WNN) analysis to leverage multiple data modalities for neighbor graph generation, clustering, integration, and data transfer. WNN analysis learns, for each cell, which data modality best predicts that cell’s identity and uses these single-cell modality weights for downstream analytical tasks. This approach allows highly accurate (*r* = 0.91) quantification of difficult-to-identify T cell subsets like mucosal-associated invariant T (MAIT) cells [87]. We anticipate that the continued publication of additional multimodal references, by integrating additional dimensions of a cell’s biological identity, will enable even more robust cell type annotations.

### Analysis of dynamic cellular processes

Viral infection can drive cells towards different states with diverse functional consequences. scRNA-seq powerfully reveals RNA abundances of cells at the time of sample collection; however, transcript abundances alone are not enough to determine drivers of infection-altered cell trajectories, which are important to elucidating disease pathogenesis. RNA velocity seeks to derive cell trajectory dynamics by leveraging explicit measurements of newly transcribed pre-mRNAs (unspliced) and mature mRNAs (spliced) in order to estimate gene splicing and degradation rates. These measurements can help infer the continuous, dynamic spectra of cell states and estimate an individual cell’s position in that spectrum as a pseudotime value (Fig. 4). Both scVelo [95] and Velocyto [96] are both tools that enable an analysis of this information. RNA velocity has limitations, including its high dependency on the k-NN graph built on the data, the cells included in the collected data, and its strong dependence on two-dimensional representations for visualization built on observed transcriptional data that do not fully capture cell-state transitions [97]. The recently developed veloViz addresses some of these limitations by incorporating RNA velocity information into 2D and 3D embeddings to better capture cellular trajectories even when intermediate cell types are missing [98].

RNA velocity has been leveraged to understand drivers of myelopoiesis and lymphopenia seen in severe COVID-19 patients. To do so, Wang et al. [99] profiled bone marrow mononuclear cells of COVID-19 patients and utilized RNA velocity to identify differences in hematopoiesis. The study found that hematopoietic stem cells from patients with severe COVID-19 demonstrated preferential differentiation trajectories towards granulocyte-monocyte progenitors and away from lymphoid progenitors, potentially underlying differences in myeloid and lymphoid cell proportions in the blood of severe COVID-19 patients.

While RNA velocity can be inferred from general scRNA-seq methods, metabolic labeling combined with scRNA-seq can enable time-resolved scRNA-seq, or tscRNA-seq, through direct experimental measurements of “new” and “old” RNA molecules to more accurately measure RNA turnover rates and infer cell-state transitions. The method, scSLAM-seq (single-cell, thiol-(SH)-linked alkylation of RNA for metabolic labeling sequencing), integrates metabolic RNA labeling and biochemical nucleoside conversion with scRNA-seq to directly measure RNA turnover on the basis of U-to-C conversion rates at the single-cell level [100]. Proof-of-concept scSLAM-seq experiments on mouse fibroblast cells infected with mouse cytomegalovirus (MCMV) showed the robustness of this experimental method to identify intermittent “bursting” kinetics (periods of transcription separated by transcriptional inactivity) of genes in response to MCMV infection. An open-source software, *dynamo*, robustly integrates RNA metabolically labeled data with scRNA-seq splicing kinetics to show an increased accuracy in RNA velocity estimates in comparison to RNA velocity analysis on solely scRNA-seq splicing data when used on a metabolically labeled human hematopoiesis scRNA-seq dataset [101].

## Analysis of viral dynamics

### Impact of viral transcript abundance on cellular responses

Using an scRNA-seq method optimized to measure virus transcripts of interest can elucidate the impact of viral presence on cell function (Fig. 4). Identification of cells containing viral reads versus “bystander” (exposed but uninfected) cells can be accomplished by including the viral genomes of interest into the genome to which scRNA-seq transcripts are aligned. Additionally, Viral-Track introduced a reference genome (curated by Stano et al. [102]) that includes over 1000 virus genomes which can be used to detect both expected and unexpected viral infections [10, 32]. This viral reference genome has been used to identify a putative SARS-CoV-2 and metapneumovirus co-infection in a severe COVID-19 patient [10]. Annotating infected versus bystander cells allows the identification of differentially expressed genes (DEGs) that correlate with viral gene presence, and has been used to identify host gene correlates of lymphocytic choriomeningitis virus (LCMV) infection of mice in vivo [10].

Unlike in typical bulk RNA-seq workflows, UMI labeling of unique transcripts prior to amplification in scRNA-seq methods allows for better estimation of the interconnectedness between viral abundance and cellular responses. Correlation methods, such as Pearson’s or Spearman’s rank correlation, can be utilized to connect intracellular viral abundance to cellular host responses. Utilizing viscRNA-seq of cells infected in vitro with either dengue or Zika virus, Zanini et al. [19] performed Spearman’s rank correlations of all host genes against viral RNA abundance to identify a conserved positive correlation between host transcripts involved in the endoplasmic reticulum (ER) unfolded protein response (UPR) and the abundance of Zika and dengue viral transcripts, relevant as both viruses’ replication and translation processes are largely restricted to the ER. In another example, Shnayder et al. [21] used scRNA-seq to show that lytic and latent human cytomegalovirus (HCMV) infections were distinguished by viral transcript abundance, but had similar host expression programs associated with a viral infection. Although the ability to correlate viral transcript abundance with host transcriptional programs is a powerful tool, it is important to keep in mind the challenges in capturing and accurately quantifying viral transcripts as discussed throughout this review.

Additionally, there can exist heterogeneity in which particular viral genes are expressed or not expressed that can impact host responses. Sun et al. [103] infected cells in vitro with influenza A virus (IAV), performed 10X-based scRNA-seq, and aligned transcriptomic data to a combined reference of both human and influenza virus (IAV) genomes. Among cells infected with IAV, the authors found substantial heterogeneity in host and viral gene expression [103]. With the IAV genome composed of 8 segments, while a majority of infected cells expressed genes from all 8 segments, others expressed transcripts from different subsets of these segments with few cells expressing transcripts from only 1–2 segments. The authors demonstrated that this heterogeneity was the result of cells expressing viral genes from a variable combination and number of IAV genome segments. For example, cells that did not express the IAV nonstructural segment (NS) demonstrated increased ISG and IFN-related transcripts—an expected outcome as IAV NS1 is known to suppress antiviral responses [2]. The identification of variable viral gene expression dynamics and their differential impact on host immune responses was directly enabled by a combination of single-cell resolution profiling and unbiased mapping of host and viral transcripts.

### Determining active replication by scRNA-seq

A challenge in scRNA-seq studies is the ability to determine whether measured viral transcripts are associated with actively replicating viral infection rather than non-replicating internalized, extracellularly bound, or ambient extracellular viral RNAs (e.g., those from cell supernatants, bound infection inoculum, virus from lysis of an infected cell, internalized via phagocytosis). Analytic methods to detect and remove contaminating ambient RNA transcripts have been developed by Kotliar et al. [23] to identify intracellular Ebola virus transcripts and by Young and Behjait [104], who developed SoupX (Fig. 4). These methods follow a similar workflow: (1) estimate the ambient RNA profile for empty droplets, (2) estimate the fraction of each cell’s transcript associated with the ambient RNA profile, (3) determine the level of ambient RNA contamination and correct the expression profile, which helps direct analyses towards cells with actively replicating RNA.

Additionally, methods including scSLAM-seq that utilize metabolic labeling of new and old RNA transcripts to infer active versus ambient transcripts [100] can help understand viral dynamics as well. Erhard et al. [100] showed that among cells infected with mouse cytomegalovirus (MCMV), scSLAM-seq was able to distinguish between “older” viral transcripts (hypothesized to be virion-associated RNA delivered to the cell) versus “newer” viral transcripts (hypothesized to be associated with actively replicating virus) to more accurately identify infected cells.

Additionally, a priori knowledge of the virus of interest can inform analyses of viral replication dynamics. In the context of DNA viruses and/or latent viruses, sequenced viral transcripts could indicate replicating and/or reactivating DNA viruses as scRNA-seq processing selects for RNA, therefore not measuring DNA viral genomes and genomically integrated virus. Authors studying herpes simplex virus (HSV)-1-infected cells, a virus that can exist in either a latent (quiescent) or lytic (active) state, were able to use measured HSV-1 transcripts as a correlate of lytic infection to identify the stepwise progression of viral gene program transcription as infection progressed and host transcripts associated with restricting viral infection [24]. In another example, utilizing the method Viral-Track to detect viral transcripts present in scRNA-seq data, researchers were able to identify human metapneumovirus (hMPV) present in a patient with severe SARS-CoV-2 infection [10]. Utilizing coverage analysis and hMPV virology, authors identified a biased nature to the transcripts (higher abundance of N, P, M, F, M2, SH, G, and lower abundance of L hMPV genes) suggesting the presence of actively replicating virus in samples at the time of collection. Another method, scCoVseq, can measure subgenomic RNA transcripts which are generally only present during active viral replication, thus having the potential to identify cells hosting actively replicating virus across coronavirus or nidovirus infections more generally [105]. Furthermore, analysis of strandedness of viral transcripts can identify potential for replicative infection: for example, if analyzing an infection by a positive-sense RNA virus, detection of negative-stranded RNA transcripts (needed as a template to build positive-sense genomic RNA to package into progeny virus particles) could indicate active replication. However, read alignment softwares such as Cell Ranger ignore antisense transcripts, so intentional design of alignment genome and processing pipelines is important to detect this information. These current analytical tools generally require a strong understanding of viral replication and viral gene expression dynamics in order to make conclusions about infection type, with the gold-standard confirmation method being plaque assay validation to prove active infection. More work is necessary to build tools across virus families to identify active versus ambient and extracellular viral RNA in scRNA-seq data.

### Deriving the differential impact of viruses within a viral quasispecies

Viruses can exist as a heterogeneous quasispecies during infection, with virions that can include a range of genomic mutations and defects. This viral genetic diversity can heterogeneously impact the responses of individual infected host cells, necessitating single-cell resolution profiling to capture the full phenotypic structure of virus-host interactions. Leitch and McLauchlan sequenced individual Huh7 cells infected with the hepatitis C virus (HCV) to demonstrate the heterogeneity in HCV quasispecies within a cell. Authors showed a cell could range from harboring only wild-type HCV sequences to containing up to four different HCV viral sequences with diverse mutations from one another, with a population of cells containing 32 different HCV sequences [15]. Authors identify differences in fitness of three HCV variants identified, which further highlights the functional impact of these viral quasispecies on the host. More work is necessary to understand the impact of viral quasispecies composition and individual variants on host antiviral responses, which may be particularly important in studying viral escape, and scRNA-seq provides a strong tool to start to answer these questions.

## Integrated views of cell signaling and communication in antiviral immunity

### Analysis of intracellular regulatory systems

**Fig. 5:**
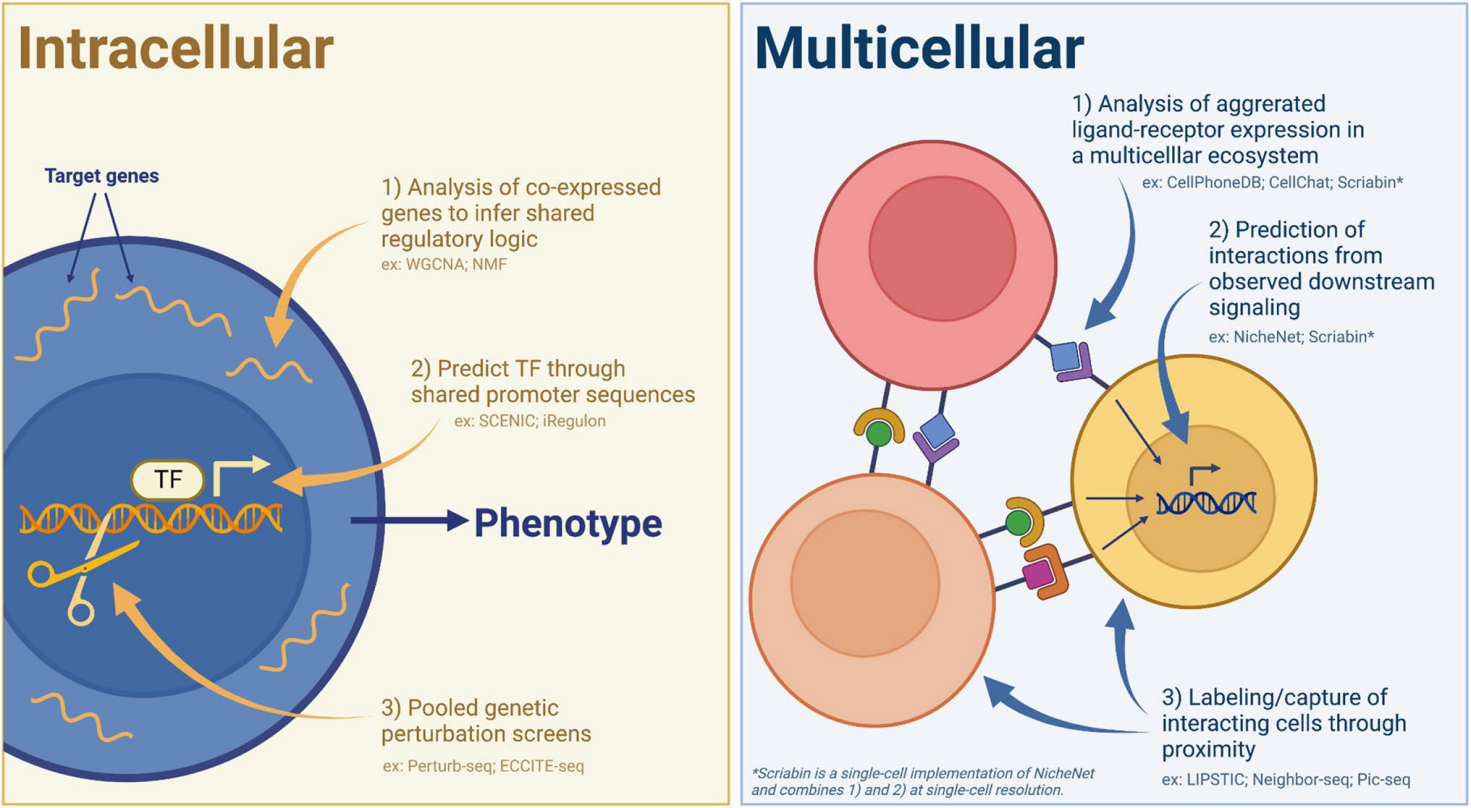
Overview of scRNA-seq analytic approaches to understanding intracellular and multicellular signaling

At its most basic level, scRNA-seq data provides a single transcriptional snapshot of cell state and identity, but it is possible to go beyond this to provide insights into intercellular regulatory logic and signaling (Fig. 5). For example, identifying groups, or modules, of genes that are co-expressed can imply orthogonal biological functionality. One of the most widespread approaches to this analytical question is weighted gene correlation network analysis (WGCNA) [106, 107]. Originally developed for bulk transcriptomic datasets, WGCNA identifies clusters of genes with a high degree of topological overlap, a measure of gene interconnectedness, between samples. WGCNA has recently been adapted to single-cell transcriptomic datasets by discovering modules of highly connected genes between individual cells rather than between separate samples [30, 108]. This approach has recently been applied to longitudinal scRNA-seq profiling of hyperacute HIV infection, where it revealed temporally coordinated and prolonged expression of gene modules associated with NK cell cytolytic activity as potentially associated with future viral control [30, 109]. Another complementary approach to WGCNA involves non-negative matrix factorization (NMF). NMF-based approaches, including consensus NMF (cNMF) [110], have recently been adapted for scRNA-seq data, where they have been applied to disentangle highly interconnected gene programs that define cell state rather than cell type. For example, cNMF has been used to identify a gene program that is associated with bacterial sepsis [111]. This same gene program has now been shown to be strongly associated with severe COVID-19 [18], and is directly inducible in hematopoietic progenitors treated with plasma from severe COVID-19 patients [112].

In addition to implying shared biological functionality, strongly co-expressed gene modules can also reflect the activity of a shared intercellular regulatory network. Many recent methods have sought to leverage genetic and epigenetic data on gene regulatory pathways to infer what transcription factors underlie observed gene modules or DEGs, thereby yielding a more integrated view of cellular phenotype. For example, SCENIC [113] and companion tool iRegulon [114] use promoter and enhancer sequences associated with each gene to predict which motifs and transcription factors are most likely to underlie observed transcriptional profiles. This approach has been used to identify STAT1/2/3 as a key putative driver of neutrophil activation in severe COVID-19 [18]. Alternatively, databases of transcriptional regulation have also been assembled from perturbation experiments, enabling the prediction of transcription factor activity from empirical measurements [115–118]. These databases have been applied to identify the activity of IRF9 in the alveolar epithelial cells of patients with severe COVID-19 [119, 120].

Recent developments in gene editing technology have enabled multiplexed genetic perturbation screens to be combined with deep single-cell transcriptomic readouts, providing approaches to directly uncover gene regulatory networks at a single-cell resolution [121–124]. These methods have recently been applied to uncover several host dependency factors and pathways for SARS-CoV-2, including the NF-κB inhibitor IκBα (NFKBIA) [125] and RAB7A, which prevents sequestration of the ACE2 receptor [126, 127]. In addition to performing functional genetic mapping of host factors, these techniques can be used to simultaneously perturb viral genetic elements. Hein and Weissman [12] have recently used Perturb-seq to map both host and viral factors that are protective or detrimental to Epstein-Barr virus (EBV) infection. They leveraged the single-cell resolution of Perturb-seq to describe a stereotyped trajectory of EBV infection that could be altered by the deletion of viral factors, but slowed or accelerated by the deletion of host factors [12]. These experiments highlight the power of integrating pooled genetic perturbations with single-cell transcriptomic readouts, providing a deep view of host and viral interactions.

### Analysis of multicellular ecosystems

In the setting of antiviral immunity, immune cells do not exert their functions in a solitary void but are rather involved in finely balanced communication networks with their microenvironment and other immune cells in order to limit viral disease. By providing a deep view of cellular phenotype at single-cell resolution, scRNA-seq datasets are well suited for the prediction of how individual cells may communicate with each other in a tissue niche (Fig. 5). The curation of ligand-receptor interaction databases has enabled the development and application of many tools to infer patterns of cell–cell communication (CCC) from scRNA-seq data [128, 129].

The most common approach to inferring CCC in scRNA-seq data is to average ligand and receptor expression values for a given cluster or cell type, use these aggregated values to predict which cells are most capable of communicating, and identify which ligand-receptor edges are most specific to communicative pathways between given cell types. These methods include CellPhoneDB [130, 131], CellChat [132], Connectome [133], NATMI [134], SingleCellSignalR [135], and iTALK [136] (reviewed by [137]). These methods have been applied to many scRNA-seq datasets profiling antiviral immune responses. For example, a recent preprint identified SARS-CoV-2-mediated induction of *CCL2* in activated interstitial macrophages as a potential mechanism to recruit specific dendritic cell (DC) subtypes through the expression of *CCR2* [138].

However, the expression of a cognate ligand-receptor pair by two cells does not demonstrate that those cells are indeed interacting or that the putative interaction impacts downstream cellular phenotype. To address this issue, NicheNet introduced a curated database linking ligand activities to target gene expression and developed a method to infer ligand activity from a set of DEGs [139]. In the setting of COVID-19, NicheNet has been applied to identify persistent IFN-α signaling in NK cells from patients with severe COVID-19 [140], IFN-γ and TNF-α as ligands driving monocyte dysfunction in post-acute sequelae of COVID-19 (PASC) [141], and IL-15 and IL-18 as macrophage-expressed ligands predicted to enhance functional activity of SARS-CoV-2 antigen-reactive CD4 and CD8 T cells [63].

A major limitation of these tools is that they operate at the level of the cell type or cell cluster and thus can obscure biologically-important heterogeneity and specificity. Our lab has demonstrated that CCC analysis methods that aggregate at the level of the cell type or cluster lose > 50% of unique CCC phenotypes in the process of agglomeration, highlighting the importance of maintaining single-cell resolution [142]. Two recent methods, NICHES [143] and Scriabin [142], present techniques to analyze CCC at near single-cell resolution. The fundamental advancement in both of these methods is the encoding of CCC information in a cell–cell matrix that measures the interaction potential of cell–cell pairs along each possible ligand-receptor edge [142, 143]. In applying Scriabin to a longitudinal dataset of SARS-CoV-2 infection [144], Scriabin revealed that uninfected bystander epithelial cells may initiate downstream inflammatory pathways through the production of *IL1B* which can act on infected cells to upregulate acute-phase reactant encoding genes involved in tissue remodeling processes [142].

Another complementary set of techniques for CCC inference are computational methods that infer which cells are communicating by identifying putative multiplets in the dataset (e.g., Neighbor-seq [145]), or by directly sequencing interacting cells (e.g., PIC-seq [146]). While this provides an additional layer of evidence for biologically-meaningful interactions, cells that have previously interacted but are no longer associated will not be detected. This latter problem has been addressed by techniques such as LIPSTIC [147] that permanently label cells that have interacted using particular ligands or receptors. However, these methods remain poorly scalable and require prior cell engineering. We anticipate that future technological developments will enable the synergy of these complementary approaches towards more comprehensive solutions for CCC analysis.

## Multimodal profiling of viral infections

### Integrated transcriptomic and genomic single-cell methods

**Fig. 6:**
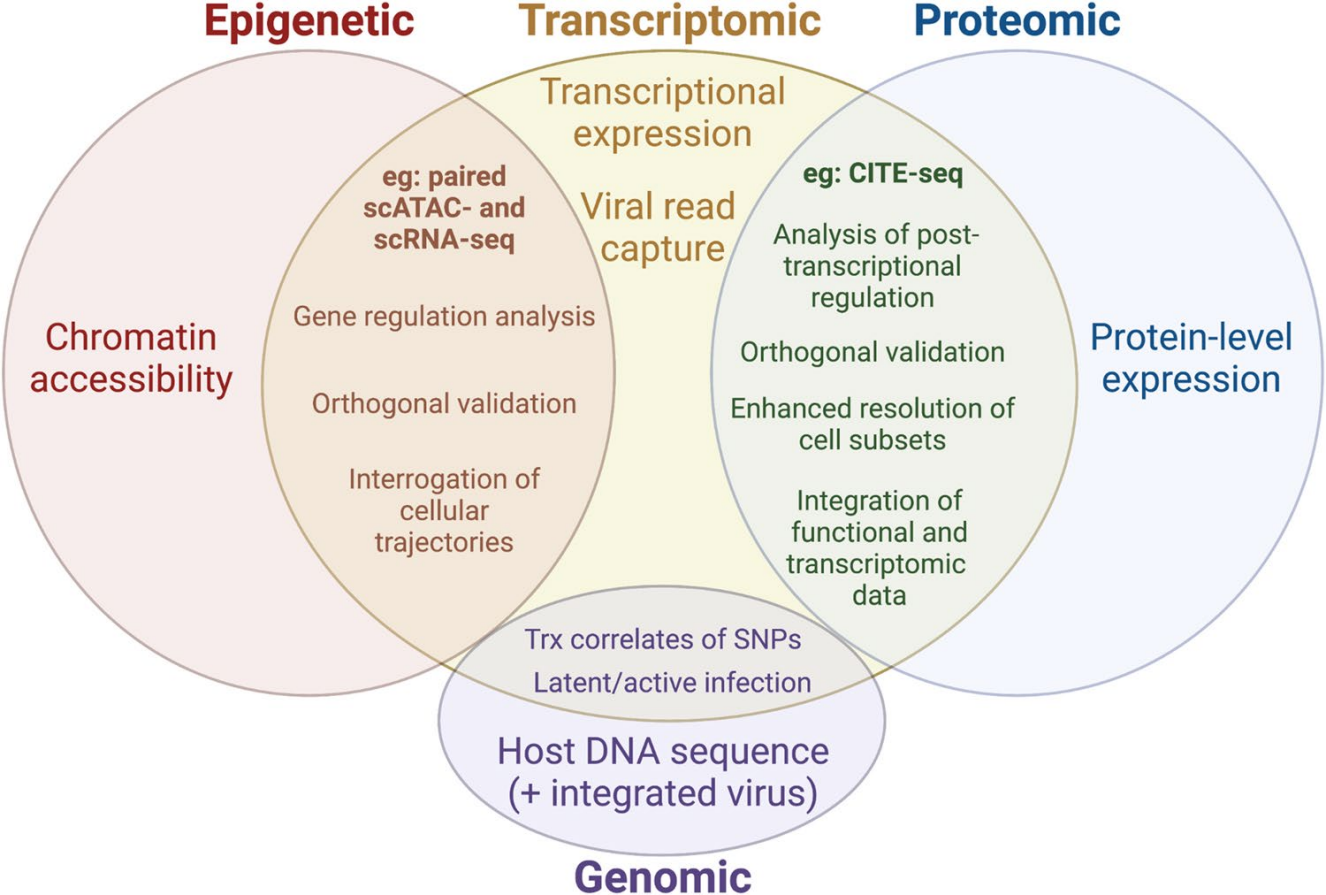
Venn diagram demonstrating the information that can be derived from epigenetic, transcriptomic, and proteomic measurements. Overlapping regions contain unique information that can be derived from integrative analyses of multiple omics in the same cell. CITE-seq, cellular indexing of transcriptomes and epitopes by sequencing; trx, transcription; scATAC-seq, single-cell resolution in assay for transposase-accessible chromatin using sequencing; SNP, single nucleotide polymorphism

Having genomic information to underlie transcriptomic changes can highlight the role of inherent genetic differences that may drive different antiviral responses and viral susceptibility (Fig. 6). Mutations in host proteins required for viral replication can alter disease susceptibility. For example, individuals homozygous for the CCR5 delta32 allele are resistant to HIV infection [148, 149], while polymorphisms of ACE2, a cellular entry receptor for SARS-CoV-2, may impact ACE2 protein expression and SARS-CoV-2 binding to potentially affecting COVID-19 pathogenesis [150]. Furthermore, associations between the HLA genotype and SARS-CoV-2 susceptibility and disease progression have been reported [151]. To simultaneously study the complexities of genomic variation on transcriptional profiles, G&T-seq (genome and transcriptome sequencing) is a method able to measure both genomic and transcriptomic information from the same cell [152]. After single-cell plating and cell lysis to release mRNA and genomic RNA, polyadenylated mRNA transcripts are physically separated from the DNA with the use of biotinylated oligo-dT primers, and both the RNA and DNA libraries are processed in parallel. Other methods for paired transcriptomic and genomic measurements of single cells include TARGET-seq [153], SIDR [154], and DR-seq [155]. DNA measurements in combination with transcriptomics can also enable an analysis of DNA virus genomic abundance as well as capture any integrated viral reads (e.g., HIV) or detect latent versus lytic viral infection (e.g., HSV1, HSV2) that may drive virus expression dynamics downstream.

### Integrated transcriptomic and proteomic single-cell methods

Technical methods have been developed to integrate scRNA-seq measurements with proteomic measurements of the same cells, such as CITE-seq [94] and REAP-seq [156]. The power of this integrated approach is the ability to correlate transcript abundance with translated protein expression—important for understanding downstream functional responses. Additionally, CITE-seq also empowers the analysis of transcriptomes within cell types defined by surface proteins, which is important as some cell types can be difficult to distinguish on transcriptomes alone (e.g., NK cells versus T cells, intermediate monocytes) (Fig. 6). A study looking at differences between hospitalized patients across moderate, severe, critical, and fatal COVID-19 severities utilized CITE-seq to measure PBMC transcriptomes and paired protein measurements across 188 cell surface proteins [157]. The paired transcriptome-protein analysis empowered the study’s ability to distinguish NK cell subtypes (CD56^hi^CD16^lo^ NK, CD56^dim^CD16^hi^ NK, CD56^lo^CD16^lo^ NK) and further identify IL-15-linked fatty metabolism and attenuated inflammation in CD56^dim^CD16^hi^ NK cells as a primary correlate of disease severity across the study group. While unpaired at the cellular level, CyTOF and scRNA-seq profiling of whole blood from the same COVID-19 patients was able to identify significantly decreased expression of activating receptors DNAM-1 and NKG2D protein in activated NK cells from severe COVID-19 samples compared with healthy, but no changes in levels of their transcripts [18], which provides an example for the importance of multimodal analyses as different conclusions could be had when focusing on one measurement for analyses.

### Integrated transcriptomic and epigenomic single-cell methods

Investigating the association between transcriptional and epigenetic variation can elucidate underlying mechanisms and regulatory features that drive immune responses to viral infections (Fig. 6). Previous research has demonstrated that exposure to different environments and vaccines can drive persistent epigenetic changes and downstream cellular responsiveness to infection and viral susceptibility. For example, work from Wimmers et al. [158] demonstrated that an adjuvanted H5N1 influenza vaccine can change the histone modification landscape of monocytes, with changes to chromatin accessibility that associate with increase expression of antiviral- and interferon-related genes and resistance to dengue and Zika virus infection [158]. There are multiple methods that can characterize both the transcriptomics and the epigenomics of a single cell and vary at what epigenomic and transcriptomic layers they can capture. One method, the 10X Chromium Single-Cell Multiome ATAC + Gene Expression kit, works by incubating nuclei suspensions with Transposases to fragment open region fragments and the kit’s Gel Beads include (a) a poly(dT) sequence to build barcoded cDNA libraries from polyadenylated nucleic mRNA as well as (b) a Spacer sequence to attached to transposed DNA fragments for the ATAC library. However, it is important to note that methods using nuclei rather than the whole cell as input into RNA-seq would be unable to detect viral transcripts from viruses that don’t use the nucleus for its life cycle (e.g., flaviviruses, coronaviruses). Another method called scM&T-seq (single-cell methylome and transcription sequencing) [159] utilizes the previously mentioned scG&T method but instead of DNA isolation for genomic sequencing, scBS-seq (single-cell bisulfite sequencing) is applied to isolated DNA to generate methylomes from the same single cells that transcriptomes are generated. A further adaptation to scM&T-seq is called scNMT-seq (single-cell nucleosome, methylation, and transcription sequencing) which adds measurement of chromatin accessibility by adapting Nucleosome Occupancy and Methylation sequencing (NOMe-seq) methods prior to BS-seq [160]. Together, all these methods can begin to bring together a deeper understanding of regulatory mechanisms and cellular trajectories driving immune responses to viral infections and the differential drivers that contribute to protective versus pathogenic responses.

### Integration and reanalysis of public scRNA-seq datasets

The expensive nature of generating scRNA-seq datasets in comparison to methods like bulk RNA-seq and flow cytometry tends to limit the number of samples run via scRNA-seq. Therefore, studies can vary in the number of samples profiled and further limits the heterogeneity of experimental conditions and sampled populations that are included. The advent of public repositories to submit scRNA-seq data and the accessibility to these resources, such as NCBI’s Gene Expression Omnibus (for processed data) and the Sequence Read Archive repositories (for raw sequencing data), make it possible for scientists around the world to download and reanalyze collected data. Xu et al. [161] reanalyzed data collected by Zanini et al. [11] of scRNA-seq of PBMCs from dengue-infected patients and employed new tools such as CellChat [132], which was developed in 2021 after the data was published in 2018, to identify cell–cell communication rewiring of PBMCs from severe dengue disease compared to control and mild dengue patients.

Additionally, a comparative understanding of infection across heterogeneity-associated disease manifestations, experimental conditions, and sampling population differences can drive a robust understanding of viral responses across included contexts. Additionally, data integration can boost sample sizes across multiple axes to power analyses. For example, a review by Tian et al. [162] integrated high-quality cells from 21 publically deposited COVID-19 scRNA-seq profiling studies of mainly PBMC and whole blood. In the end, authors collectively analyzed 3.2 million cells from COVID-19 patients from various demographics and disease severities to identify cell type correlates of COVID-19 pathogenesis [162]. Integrated analyses with non-viral diseases are also powerful: Reyes et al. [112] comparatively analyzed scRNA-seq of PBMCs they collected from patients with bacterial sepsis and public COVID-19 PBMC scRNA-seq data to identify shared monocyte transcriptional responses during severe disease, marked by reduced MHC-II transcripts (important for antigen presentation) and increased expression of S100A8 (implicated in the development of myeloid-derived suppressor cells). Further understanding viral infection responses comparatively with other diseases and conditions (including vaccination) is an important avenue for exploration to better understand protective/pathogenic mechanisms.

## Conclusions and future directions

scRNA-seq overcomes some of the limits of bulk sequencing methods to measure the heterogeneity of viral dynamics and cellular responses in relation to one another (Fig. 1). With new scRNA-seq methods able to integrate additional measurement technologies, an incredible amount of information can be uncovered from a single sample. Multimodal methods that integrate transcriptomics with other biological information are an important frontier to gaining a deeper understanding of the complexities of human antiviral responses. Additionally, integrated analyses of data, not just within one type of viral infection, but also across various other viral infections, vaccine responses, and conditions (e.g., sepsis) might also add to our understanding of viral pathogenesis through an understanding of the complexities of cellular responses. Public availability of scRNA-seq data paired with constantly developing data analysis tools allows a deeper understanding of viral-host dynamics, thereby allowing these data to continue contributing scientific knowledge far beyond data collection. It remains important to continue scRNA-seq studies across diverse viruses in the context of diverse cell types and populations to gather a holistic understanding of disease pathogenesis. Importantly, all methods discussed have their various pros and cons: when designing an scRNA-seq study uncovering virus-host responses, it is crucial to understand the study’s goals and limitations (e.g., virus type, cell types of interest, sample number, budget) in order to decide on a method that can optimally measure the information of interest. For example, while many studies focus on blood, a number of viruses target specific tissues and may not be present in the blood, thus limiting the power of discussed methods to optimize virus quantification and dynamics. Therefore, utilizing prior information regarding viral tropism and disease dynamics to inform scRNA-seq study and method design can go a long way to obtaining high-information data.

There also remain broad limitations on the use of scRNA-seq technology. For example, the techniques discussed in this review require the sample input to be a single-cell suspension; therefore, no spatial information is captured, as solid tissues must be dissociated prior to analysis. This results in the omission of important information in solid tissue infections (e.g., lung infection in SARS-CoV-2) though is less of an issue for blood-borne pathogens. Newer methods such as spatial barcoding and high-plex RNA imaging seek to remedy this limitation [163, 164]. Additionally, although the throughput of scRNA-seq methods has vastly improved since the advent of these technologies, the maximum cell number that can be used in these workflows is still far below the number of cells that can be processed by single-cell proteomic methods such as flow cytometry and CyTOF. This is due to both technological limitations of scRNA-seq methods as well as cost prohibition, as high-throughput sequencing is quite expensive.

**Fig. 7:**
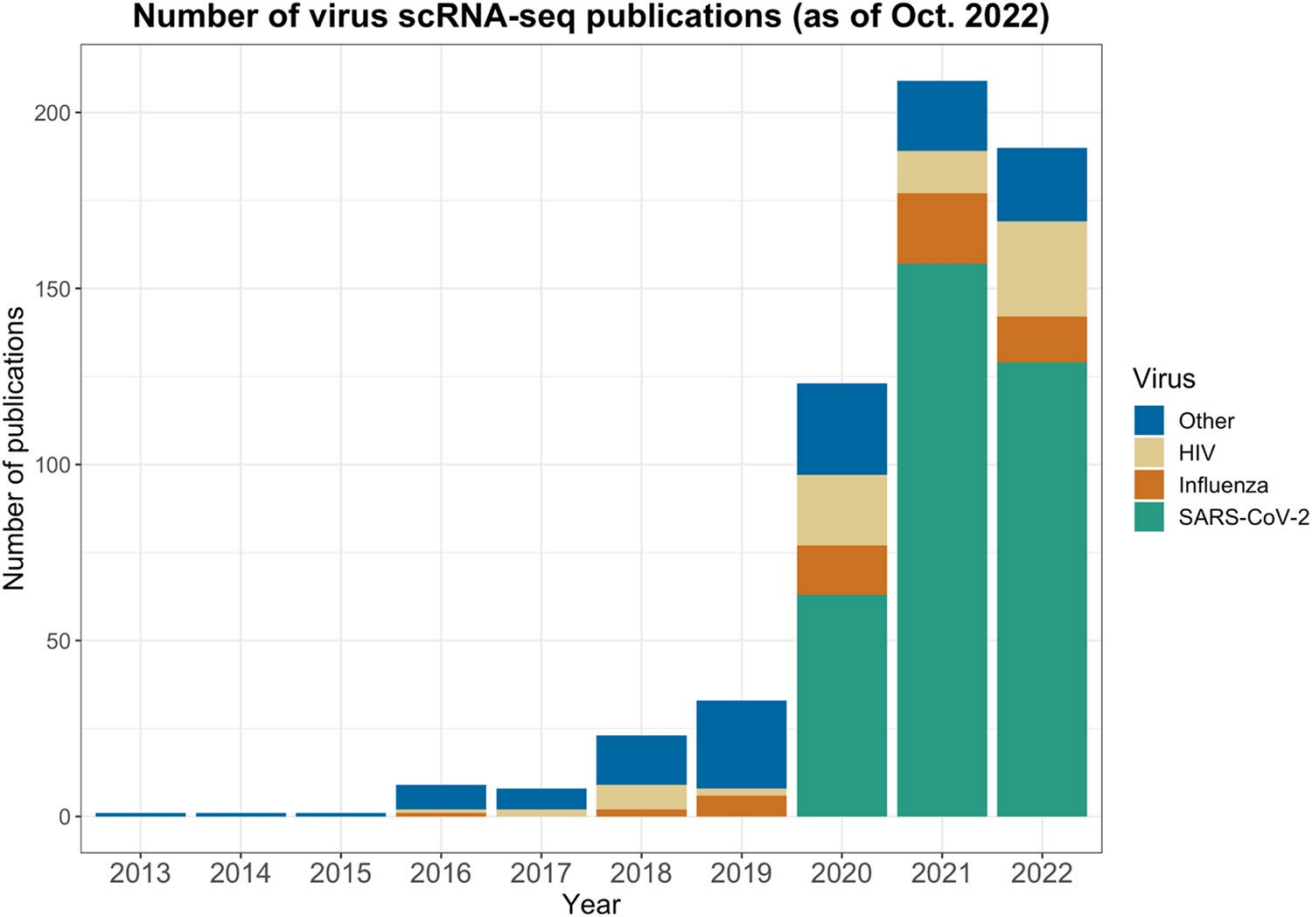
Virus-related scRNA-seq publications from 2013 to October 2022. SCOPUS database search for publications associated with “scRNA-seq” and “viruses,” with publications relating to HIV, influenza, and SARS-CoV-2 highlighted within the identified total

We have learned a lot about antiviral immunity with the advent of scRNA-seq technology—and there is still more to learn. There is a wealth of scRNA-seq publications on SARS-CoV-2 (Fig. 7), and we must extend scRNA-seq applications across other viral diseases, including those identified by WHO as priority diseases [165]. A proactive rather than reactive application of scRNA-seq methods to a broader range of viral infections will allow us to better understand protective and pathogenic cellular responses to viruses to be better equipped to manage current and emerging viral diseases.
